# Molecular identification of *Phlebotomus kandelakii* apyrase and assessment of the immunogenicity of its recombinant protein in BALB/c mice

**DOI:** 10.1038/s41598-023-36037-z

**Published:** 2023-05-30

**Authors:** Shima Fayaz, Abbasali Raz, Fariborz Bahrami, Pezhman Fard-Esfahani, Parviz Parvizi, Soheila Ajdary

**Affiliations:** 1 Department of Immunology, Pasteur Institute of Iran, Tehran, Iran; 2 Department of Biochemistry, Pasteur Institute of Iran, Tehran, Iran; 3 Malaria and Vector Research Group (MVRG), Biotechnology Research Center (BRC), Pasteur Institute of Iran, Tehran, Iran; 4 Department of Parasitology, Pasteur Institute of Iran, Tehran, Iran

**Keywords:** Hydrolases, Parasitic infection, Protein vaccines

## Abstract

Sand fly salivary proteins have immunomodulatory and anti-inflammatory features; hence, they are proven to perform important roles in the early establishment of *Leishmania* parasite in the vertebrate host. Among them, salivary apyrase with anti-hemostatic properties has a crucial role during the blood meal process. In the present study, a Genome-Walking method was used to characterize a full-length nucleotide sequence of *Phlebotomus (P.) kandelakii* apyrase (Pkapy). Bioinformatics analyses revealed that Pkapy is a ~ 36 kDa stable and hydrophilic protein that belongs to the *Cimex* family of apyrases. Moreover, recombinant proteins of Pkapy and *P. papatasi* apyrase (Ppapy) were over-expressed in *Escherichia coli* BL2 (DE3) and their antigenicity in BALB/c mice was evaluated. Dot-blot and ELISA results indicated that both recombinant apyrases could induce antibodies in BALB/c. Moreover, a partial cross-reactivity between Pkapy and Ppapy was found. In vitro stimulation of splenocytes from immunized mice with the recombinant proteins indicated cross-reactive T cell proliferative responses. Cytokine analysis revealed significant production of IFN-γ (*p* < 0.001) and IL-10 (*p* < 0.01) in response to Pkapy. In conclusion, the full-length nucleotide sequence and molecular characteristics of Pkapy were identified for the first time. Immunologic analyses indicated that Pkapy and Ppapy are immunogenic in BALB/c mice and show partial cross-reactive responses. The immunity to Pkapy was found to be a Th1-dominant response that highlights its potential as a component for an anti-*Leishmania* vaccine.

## Introduction

Phlebotomine sand flies are blood-feeding insects that act as vectors of different species of *Leishmania* (*L.*) parasites in the Old World. During blood-feeding, the parasites are inoculated at the biting site along with the sand fly saliva. The saliva contains pharmacologically-active components to facilitate the acquisition of blood meals and exhibits immunomodulatory properties^1,2^. These compounds also support the survival and multiplication of the parasites and facilitate disease progression^2–4^. It has been shown that pre-exposure to non-infected sand flies or pre-immunization with salivary gland lysate (SGL) might protect mice, hamsters, dogs, and non-human primates against a few *Leishmania* species^3^. Both humoral and cellular immune responses against different salivary components of sand fly have been detected. Generally, anti-saliva antibody response and DTH/T cell proliferative responses are considered markers of exposure to sand fly bites and protection against leishmaniases, respectively^4^.

Several sand fly species including *Phlebotomus (P.) kandelakii*, *P. perfilewi transcaucasicus,* and *P. tobbi* have been identified in different visceral leishmaniasis (VL) foci in Iran. *P. kandelakii* is one of the most prevalent vectors in the North-East and North-West regions of the country where natural infection of this species by *L. infantum* has been reported^5,6^. Although several reports have been published related to the composition and immunogenicity of the salivary proteins of at least 13 sand fly species^4^, not much information is available about the composition of *P. kandelakii’s* saliva.

The composition and immunogenicity of the salivary proteins are species-specific. Volf et al*.* have reported important differences in the antigen components of the SGL from *P. papatasi*, *P*. *perniciosus,* and *P*. *halepensis*. Besides, they have indicated that sera from mice bitten by *P*. *papatasi* cross-react with the homologous SGL but not with SGL from the other two species^7^. Therefore, exploring the salivary antigens from different *Leishmania* vectors is a main priority for the researchers.

Apyrases are enzymes that hydrolyze ATP and ADP to AMP and orthophosphate; thereby, can destroy these important stimuli of platelet aggregation and inflammation at the biting site^8^. Apyrases of the blood-feeding insects are divided into three families. The *Cimex* family of apyrases functions exclusively with Ca^2+^ while the other two families require either Ca^2+^ or Mg^2+^ for their actions^9,10^. The *Cimex* family of apyrases is among the most prevalent proteins in sand fly saliva and counteracts the host's hemostatic system by inhibiting the blood coagulation cascade, platelet aggregation, and vasoconstriction^2,11,12^. Apyrases from different sand fly species elicit antibody responses in various hosts including *P. papatasi* apyrase (Ppapy) in humans and mice^13,14^, *P. perniciosus* apyrase in mice, rabbits, and dogs^15–18^, *P. tobbi* apyrase in rabbit^19^, and *P. orientalis* apyrase in domestic animals^20^. Altogether, the results of these studies have confirmed that apyrases belong to the most antigenic salivary proteins and can be recognized by sera of the repeatedly-bitten hosts^16^. Therefore, anti-apyrase antibodies can be considered a promising biomarker of sand fly exposure or even a risk factor for *Leishmania* transmission among the hosts. Besides, cell-mediated immune responses against *P. ariasi* apyrase and *P. papatasi* apyrase have been documented^21–23^.

The sequences of apyrases from different species of sand flies have so far been reported^11,22–24^; however, only partial information has been available on *P. kandelakii* apyrase (Pkapy) gene. In the present study, we aimed for the first time to identify a full-length nucleotide sequence of Pkapy from Iran. Besides, the amino acid sequence of Pkapy was analyzed by in silico approaches. The recombinant protein was then expressed in *Escherichia coli* and its antigenicity in BALB/c mice was evaluated. Since *P. papatasi* is the main and proven vector of *L. major* in different parts of Iran^23^, we also compared the in-silico findings of Pkapy and Ppapy and the immunogenicity and potential cross-reactivity of recombinant Pkapy and Ppapy were investigated in BALB/c mice. Finally, the cytokine profile in response to Pkapy was evaluated.

## Results

### Characterization of *P. kandelakii apyrase* gene

A Genome-Walking procedure was performed to determine the full-length sequence of a partially known part of *P. kandelakii* apyrase. During the synthesis stage of ssDNA using gradient PCR, the lowest temperature with no amplicon was 58 °C. Therefore, this temperature was considered the annealing temperature for performing the first step of the procedure (Table 1b). For the determination of the downstream target sequence, the second nested PCR products of F-GSPc and UAP-N2 primers were analyzed on 1.5% agarose gel. Different amplicons were observed in all GW tubes (Supplementary Fig. S1a). To evaluate the upstream of the middle part of the gene, the second nested PCR products of R-GSPc and UAP-N2 primers were run among which a few fragments were related to GWA, GWC, and GWF (Supplementary Fig. S1b). Based on comparisons of the lengths of apyrase genes in different sand fly species, the size of Pkapy was estimated to be approximately 400 bp at the 3′-end and 150 bp at the 5′-end. Therefore, the amplicons of more than 400 bp (i.e., 3 amplicons in GWA, 1 amplicon in GWF, and 1 amplicon in GWG) were selected for the downstream sequence assessment (Additional file 1: Fig. S1a), and amplicons of more than 150 bp (i.e., 2 amplicons in GWA for upstream sequence determination; Additional file 1: Fig. S1b) were selected and extracted from the agarose gel and were subjected for TA cloning and nucleotide sequencing. The retrieved sequences were revealed to belong to the apyrase gene family by nucleotide BLAST. These sequences were assembled onto the middle part sequence using the overlapping regions. Consequently, the full assembled sequence of *Pkapy* gene was submitted to GenBank (Accession N^o^.: MN893300).

**Table 1:** List of Genome-walking primers (a) and PCR programs (b).

(a) Primers		
Experiments	Primer name	Sequence (5′ to 3′)
Gene-specific primers	F-GSPa	CGGCGGAGATTTAATTCCATGGGTGATTCTCTC
	F-GSPb	CGAATGGGCGACAGTTAAGGATG
	F-GSPc	CCTCTGGGTGAAGGAAATCGAC
	R-GSPa	AATCACCCATGGAATTAAATCTCCGCCGTG
	R-GSPb	ACAACTCGGACAGTTCGGCTC
	R-GSPc	CGCGTGAAGTAATGAAGATTCTCACTC
Genome Walking primers	GWA	GATCAGGCGTCGCGTACCTCNNCTACTG
	GWB	GATCAGGCGTCGCGTACCTCNNCTACT
	GWC	GATCAGGCGTCGCGTACCTCNNCTAC
	GWD	GATCAGGCGTCGCGTACCTCNNCACGCA
	GWE	GATCAGGCGTCGCGTACCTCNNCACGC
	GWF	GATCAGGCGTCGCGTACCTCNNCACG
	GWG	GATCAGGCGTCGCGTACCTCNNGAGAC
	UAP-N1	CCTGTGAGCAGTCGTATCCACCGATCAGGCGTCGCGTACCTC AGGCGTCGCGTACCTC
	UAP-N2	CCTGTGAGCAGTCGTATCCAC

(b) PCR program		
Steps	Program	
First step	95 °C 5 min; (95 °C 30 s, 58 °C 30 s, 72 °C 2 min) 35 cycle; 72 °C 10 min	
Second step	(94 °C 4 min, 38 °C 1 min, 72 °C 1 min) 10 cycle; 72 °C 5 min; (94 °C 30 s, 65 °C 30 s, 72 °C 3 min, 94 °C 30 s, 65 °C 30 s, 72 °C 3 min, 94 °C 30 s, 40 °C 1 min, 72 °C 3 min) 7 cycle; 72 °C 15 min	
Third & fourth step	95 °C 5 min; (95 °C 30 s, 58 °C 30 s, 72 °C 3 min) 35 cycle; 72 °C 30 min	

### In silico findings and predictions

As indicated in Table 2 and according to the performed computations, physicochemical parameters, namely Mw and pI of Pkapy in the mature form were predicted to be 35.2 kDa and 9.2, respectively. The protein was composed of 309 amino acids, containing 35 negatively-charged (Asp + Glu), and 43 positively-charged residues (Arg + Lys). Based on the aliphatic and instability indices (79.55 and 18.48, respectively), Pkapy was predicted to be a stable protein. Moreover, the grand average of hydropathicity (GRAVY) of the predicted protein was − 0.49 which indicates its hydrophilic nature. Prediction of *N*- and *O*-glycosylation sites in Pkapy revealed that N247 is susceptible to *N*-glycosylation while there is no *O*-glycosylation site in this molecule (Table 2).

**Table 2:** Physicochemical parameters and the secondary structure predictions of Pkapy and Ppapy.

Physiochemical properties and the secondary structure predictions^a^	Pkapy^b^	Ppapy^c^
Number of amino acids	309	315
Molecular weight	35,196.08	38,856.06
Theoretical PI	9.2	8.93
Instability index	18.48	20.1
Estimated half-life	> 10 h (*E. coli*, in vivo)	> 10 h (*E. coli*, in vivo)
Aliphatic index	79.48	82.67
GRAVY	− 0.493	− 0.484
Alpha helix	7.77% (24 aa)	5.71% (18 aa)
Extended strand	39.81% (123 aa)	39.68% (125 aa)
Random coil	52.43% ((162 aa)	54.60% (172)

^a^The signal peptides were excluded for the secondary structure predictions.

^b^*P. kandelakii* apyrase.

^c^*P. papatasi* apyrase.

The secondary structure analysis of Pkapy indicated that the protein was made up of alpha-helix (7.77%), extended strand (39.81%), and random coil (52.43%). Since 162 of 309 amino acid residues were found to be localized to the random coil, this would probably be the main secondary structure of Pkapy (Table 2). Physicochemical properties and secondary structure prediction of Ppapy are also presented in Table 2. Signal peptide cleavage sites were predicted for Ppapy and Pkapy amino acid sequences between residues 21–22 and 20–21 with 0.9614 and 0.9468 probabilities, respectively.

**Figure 1:**
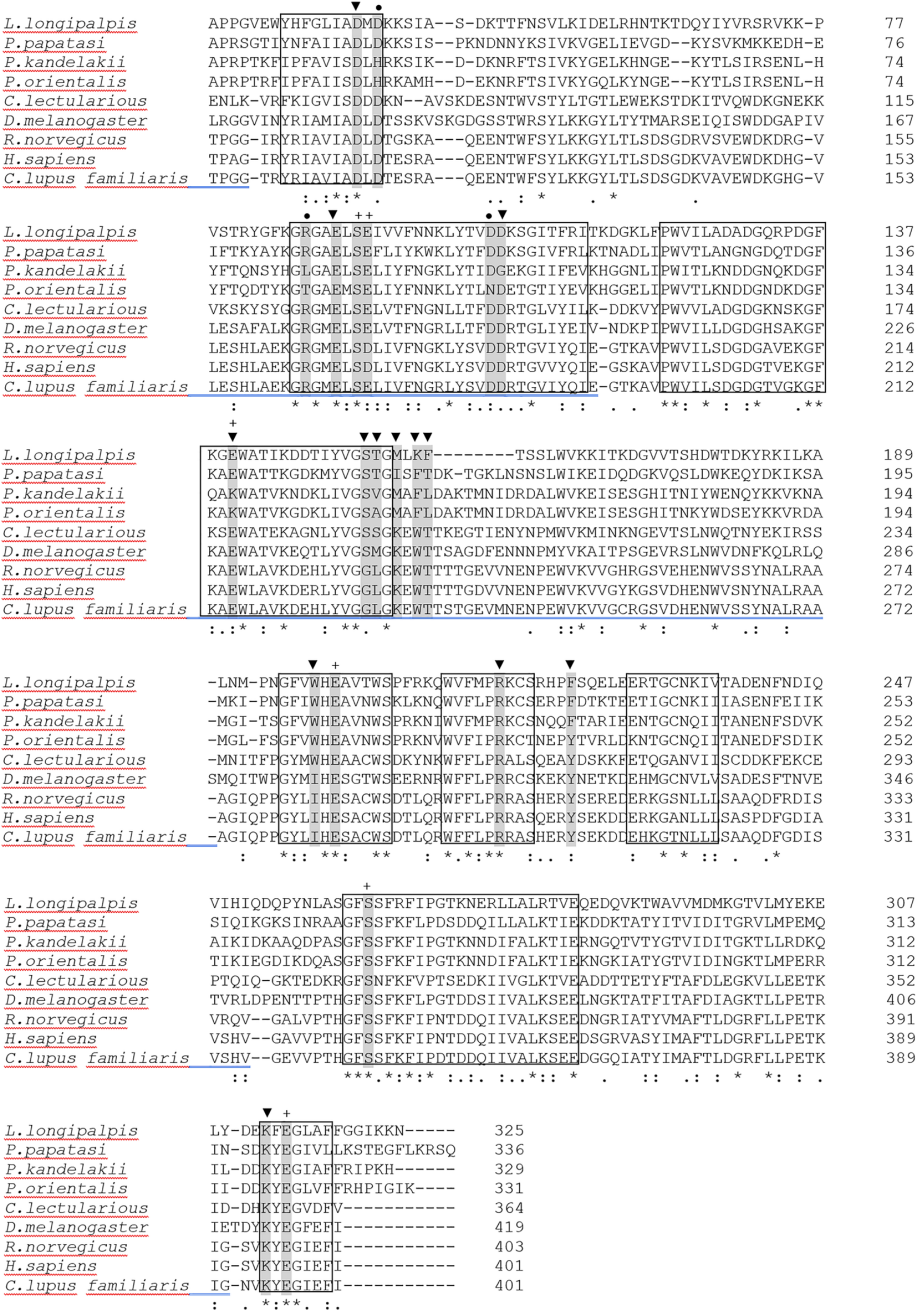
Protein sequence alignment of the *Cimex* family of salivary apyrases of *L. longipalpis* (AAD33513.1), *P. papatasi* (AAG17637.1), *P. kandelakii* (QNG40038.1), *P. orientalis* (AGT96454.1) and *Cimex (C.) lectularious* (AAD09177.1), *Drosophila (D.) melanogaster* (CAL26008.1) and CAN from *Rattus (R.) norvegicus* (AJ312207), Homo (H.) sapiens (Q8WVQ1.1), and *Canis (C.) lupus familiaris* (XP_005624095.1). Eight highly conserved sequence domains are indicated in the box. Filled triangles (▼) and pluses (+) signify important residues for binding nucleotide and Ca^2+^ in H-CAN, respectively. Filled circles (●) imply essential residues for nucleosidase activity in H-CAN. (*), (:) and (.) symbols below the alignment denote fully conserved residues, residues with strong, and weak similarity, respectively.

Protein sequence alignment comparison between Pkapy (QNG40038.1) with apyrases from a few sand fly species and other related sequences is indicated in Fig. 1. The comparison of the critical residues between Pkapy and human calcium-activated nucleotidase **(**H-CAN) demonstrated that the targeted residues are conserved to some extent (Fig. 1). This alignment revealed that 4 of the 6 residues important for Ca^2+^ binding (i.e., S168, E284, S345, E396), 4 of the 13 residues important for binding to nucleotides (i.e.,S168, E284, S345, E396), and 1 of the 3 residues essential for nucleotidase activity in H-CAN (i.e., D181) were conserved in Pkapy (Fig. 1). The 8 highly conserved regions among apyrases from vertebrates and invertebrates species, which had been introduced by Failer et al. were also conserved in Pkapy^25^. The amino acid homology and the similarity percentages between Ppapy and Pkapy proteins were 48.2% (163/338) and 68.0% (230/338), respectively.

### The tertiary structure predictions and superimpositions

**Figure 2:**
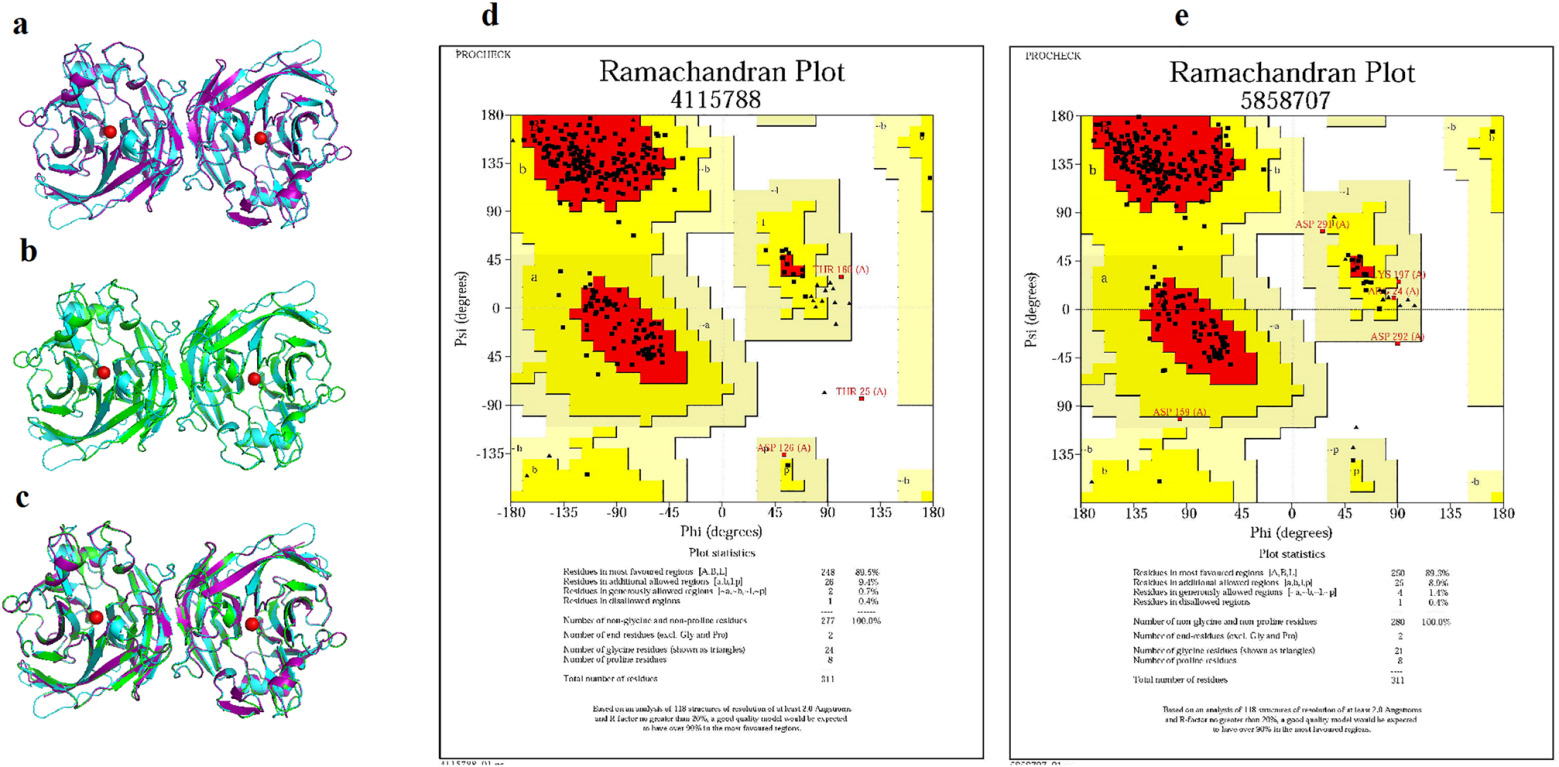
Structural analysis of the predicted models and H-CAN (as a reference molecule). Structural superimposition of (**a**) Pkapy (purple) and H-CAN (blue), (**b**) Ppapy (green) and H-CAN (blue), (**c**) Pkapy (purple)*,* Ppapy (green) and H-CAN (blue). Ca^+2^ is indicated in red. Ramachandran plot of Pkapy (**d**) and Ppapy (**e**).

Prediction of the tertiary structure of Pkapy and Ppapy using SWISS-MODEL according to the homology modeling and QMEAN and GMQE scores revealed that both aforementioned apyrases have a close topology to homo-dimer of the human calcium-activated nucleotidase (H-CAN; accession N^o^.: 2H2N PDB). Thus, H-CAN was considered as the reference molecule in superimposition and to find the counterparts of the structurally-important residues. The structural superimpositions of Pkapy and Ppapy with H-CAN revealed that they are very similar in structure, especially in critical residues and functional domains (Fig. 2a–c). Notably, a comparison of RMSDs of Pkapy and Ppapy with the reference molecule indicated that the subtraction RMSDs values of the reference and the target molecules at specific positions are less than 0.5 Å which shows the backbone similarity of the reference and Pkapy and Ppapy molecules (Table 3). In addition, analysis of the torsional angles (Ramachandran plot) to validate the 3D modeled structures of Pkapy and Ppapy showed 89.5% and 89.3% of the residues are in the favored regions, respectively (Fig. 2d,e). The superimpositions and comparisons of the critical residues of the active site revealed that the targeted residues are completely similar to their counterparts.

**Table 3:** RMSD values of the structurally important residues of Pkapy and Ppapy to the 2H2N human calcium-activated nucleotidase (CAN) as a reference.

Protein	Amino acid	position	RMSD (Å)	Importance sequence in CAN
Pkapy^a^	S, E, K, E, S, E	89, 90, 137, 205, 268, 319	0.264, 0.154, 0.296, 0.173, 0.431, 0,366	Ca^2+^ binding
Ppapy^b^	S, E, E, E, S, E	91, 92, 139, 206, 269, 320	0.220, 0.269, 0.105, 0.126, 0.190, 0.277	
Pkapy	D, E, G, k, S, V, M, F, L, W, R, F, K	35, 87, 103, 137, 150, 151, 153, 155, 156, 203, 221, 228, 317	0.182, 0.254, 0.250, 0.296, 0.132, 0.187, 0.561, 0.252, 0.397, 0.205, 0.215, 0.190, 0.306	Nucleotide binding
Ppapy	D, E, D, E, S, T, I, F, T, W, R, F, K	36, 89, 105, 139, 152, 153, 155, 157, 158, 204, 222, 229, 318	0.372, 0.221, 0.348, 0.105, 0.089, 0.176, 0.202, 0.127, 0.153, 0.115, 0.137, 0.251, 0.163	
Pkapy	H, D, L	37, 102, 84	0.151, 0.344, 0.160	Nucleotidase activity
Ppapy	D, D, R	38, 104, 86	0.100, 0.122, 0.156	

^a^*P. kandelakii* apyrase.

^b^ *P. papatasi* apyrase.

### Prediction and comparison of B cell epitopes of Pkapy and Ppapy

The linear B cell epitopes of Pkapy and Ppapy were predicted using Bepipred 2.0 server (Table 4). Based on the scores of the default thresholds, 8 and 10 linear sequences were designated as B cell epitopes of Pkapy and Ppapy, respectively (Additional file 2: Fig. S2). The profiles of the antigenic peptides were very similar together and were located in the same topological position in the first structure of the two proteins.

**Table 4:** Linear B cell epitopes of Pkapy and Ppapy.

Pkapy^a^				Ppapy^b^			
Linear Epitope^c^	Position^d^	Sequence	Length	Linear Epitope^c^	Position^d^	Sequence	Length
KLE1	18–25	RKSIKDDK	8	PLE1	17–26	DKKSISPKND	10
				PLE2	48–56	KMKKEDHEI	9
KLE2	57–67	TQNSYHGLGAE	11	PLE3	58–69	TKYAYKGRGAEL	12
KLE3	106–114	DDGNQKDGF	9	PLE4	108–114	NGDQTDG	7
KLE4	135–147	FLDAKTMNIDRDA	13	PLE5	134–148	ISFTDKTGKLNSNSL	15
KLE5	159–174	ITNIYWENQYKKVKNA	16	PLE6	158–174	KVQSLDWKEQYDKIKSA	17
KLE6	204–216	SNQQFTARIEENT	13	PLE7	204–216	SERPFDTKTEETI	13
KLE7	235–247	KIDKAAQDPASGF	13	PLE8	233–244	SIQIKGKSINRA	12
KLE8	287–298	LLRDKQILDDKY	12	PLE9	287–296	LMPEMQINSD	10
				PLE10	308–312	EGFLK	5

^a^*P. kandelakii* apyrase.

^b^*P. papatasi* apyrase.

^c^KLE, *kandelakii* apyrase linear epitope; PLE, *papatasi* apyrase linear epitope.

^d^The signal peptides were excluded for the linear epitopes predictions.

The discontinuous B cell epitopes were estimated using ElliPro, based on the 3D structure of the proteins. The protrusion index (PI) of the residues was calculated to show the residues’ solvent accessibility. Higher scores were determined as larger solvent accessibility of the residues. The PI values (above the default 0.5 scores) of discontinuous B cell epitopes for Pkapy and Ppapy are reported in Supplementary Table S1. Seven discontinuous B cell epitopes with similar topological positions for each protein were predicted.

### Expression and purification of recombinant Ppapy and Pkapy

**Figure 3:**
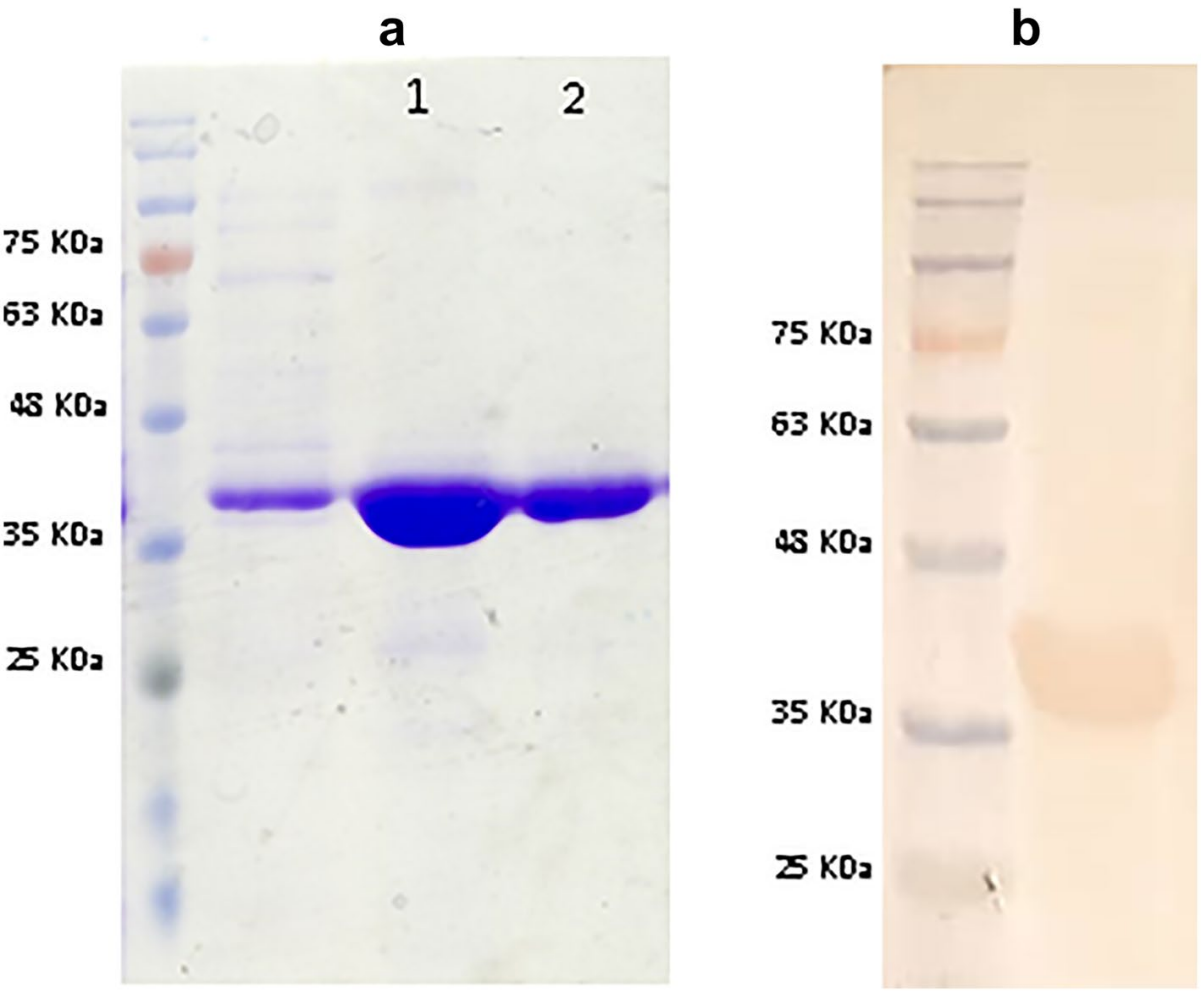
(**a**) SDS-PAGE gel stained with Coomassie Brilliant Blue. Ni–NTA-purified Pkapy (lane 1) and Ppapy (lane 2). (**b**) Western blotting of Pkapy incubated with anti-His antibody.

The recombinant Ppapy and Pkapy proteins were successfully expressed in *E. coli* and a high level of purification was achieved by Ni–NTA affinity chromatography. SDS-PAGE and Western blotting of the purified recombinant Ppapy and Pkapy indicated distinct bands with ~ 39 kDa as the expected Mw of the recombinant proteins (Fig. 3a,b, Supplementary Fig. S3).

### Antibody responses

**Figure 4:**
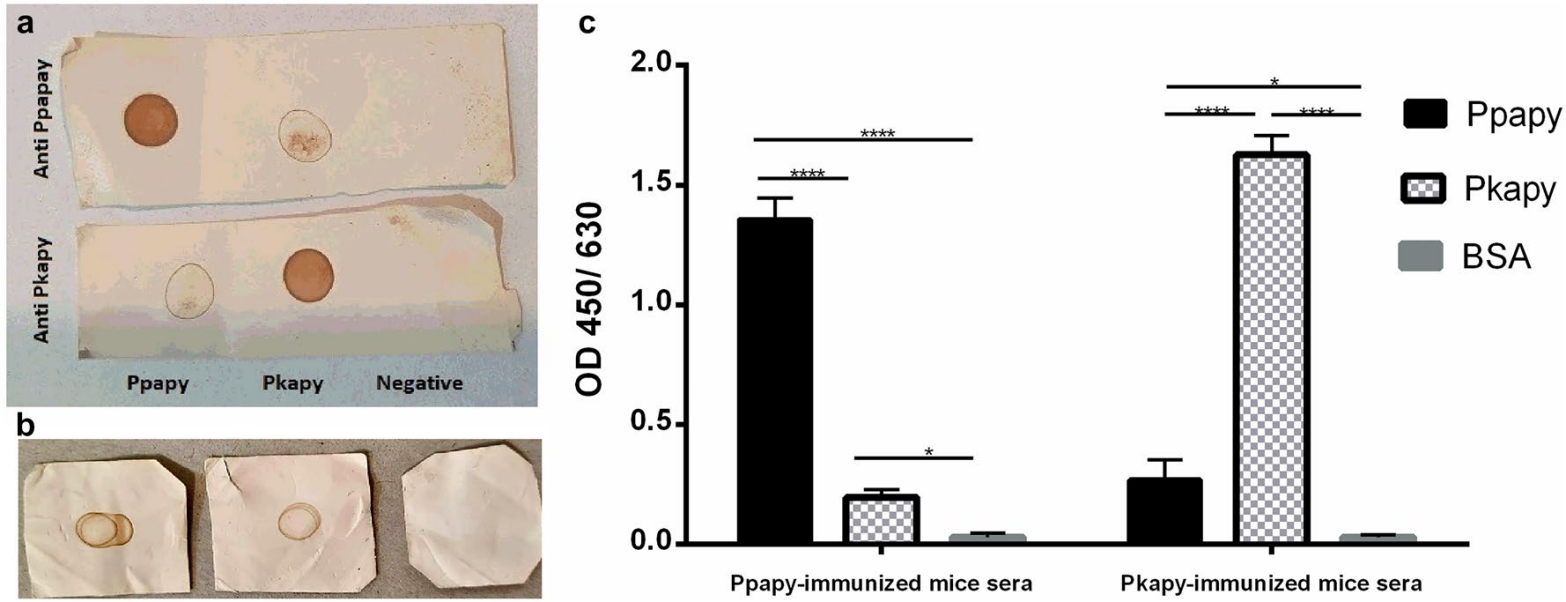
Anti-apyrase antibody analysis. (**a**) Ppapy, Pkapy, and BSA as a negative control dotted on nitrocellulose membrane and treated with anti-Pkapy and anti-Ppapy sera; (**b**) SGL from *P. papatasi* were dotted on nitrocellulose membrane and treated with sera from mice that have been immunized with recombinant Ppapy or Pkapy, normal mice sera were used as control; (**c**) Levels of serum antibodies in the mice (n = 4 in each group) immunized with Ppapy and Pkapy.; Pkapy, Ppapy or BSA were coated in the 96-well ELISA plates. Each bar indicates mean + SD of ODs, (*p < 0.05; ****p < 0.0001).

The antibody responses to and cross-reactivity between the recombinant Ppapy and Pkapy were investigated. Dot-blot results indicated that both proteins were immunogenic in BALB/c mice and induced antibodies against respective recombinant proteins. Besides, the antibodies generated in Ppapy- or Pkapy-immunized mice sera could react with both Ppapy and Pkapy but not with the control (BSA; Fig. 4a). To verify whether the recombinant proteins have conserved their immunogenic properties after expression in a prokaryote system, SGL from *P. papatasi* was spotted on a nitrocellulose membrane and the reactions with sera containing anti-Ppapy, and anti-Pkapy antibodies and normal mouse serum (as a negative control) were tested. Notably, antisera produced against the recombinant apyrases reacted with SGL from *P. papatasi* (Fig. 4b), indicating that antibodies against both recombinant proteins could react with native apyrase from *P. papatasi*. The ELISA results confirmed the immunogenicity, and cross-reactivity of the antibody responses in mice immunized with Ppapy and Pkapy. Sera from Ppapy-immunized mice reacted with Ppapy protein significantly stronger, compared to Pkapy and BSA (*p* < 0.0001). Likewise, sera from Pkapy-immunized mice reacted with Ppapy protein significantly stronger compared to Ppapy and BSA (*p* < 0.0001). The significantly higher reactivity of anti-Ppapy with Pkapy, and anti-Pkapy with Ppapy compared to reactivity with BSA (*p* < 0.05) was indicative of the specificity of the antigen–antibody reaction. The mean OD value for reactivity of anti-Ppapy sera with Ppapy protein was 1.4 while for the same amount of Pkapy-coated-wells, the mean OD value was 0.2. On the other hand, the OD values for a reaction between anti-Pkapy sera and Pkapy and Ppapy proteins were 1.6 and 0.3, respectively (Fig. 4c). These results demonstrated an average of 18.8% reactivity of anti-Pkapy sera with Ppapy protein while anti-Ppapy sera showed 14.4% reactivity with Pkapy protein. The wells coated with BSA did not show any reactivity with none of the anti-Pkapy or anti-Ppapy sera. The ELISA results confirmed the presence of a low-level cross-reactivity between Pkapy and Ppapy.

### T cell proliferation and cytokine assay

**Figure 5:**
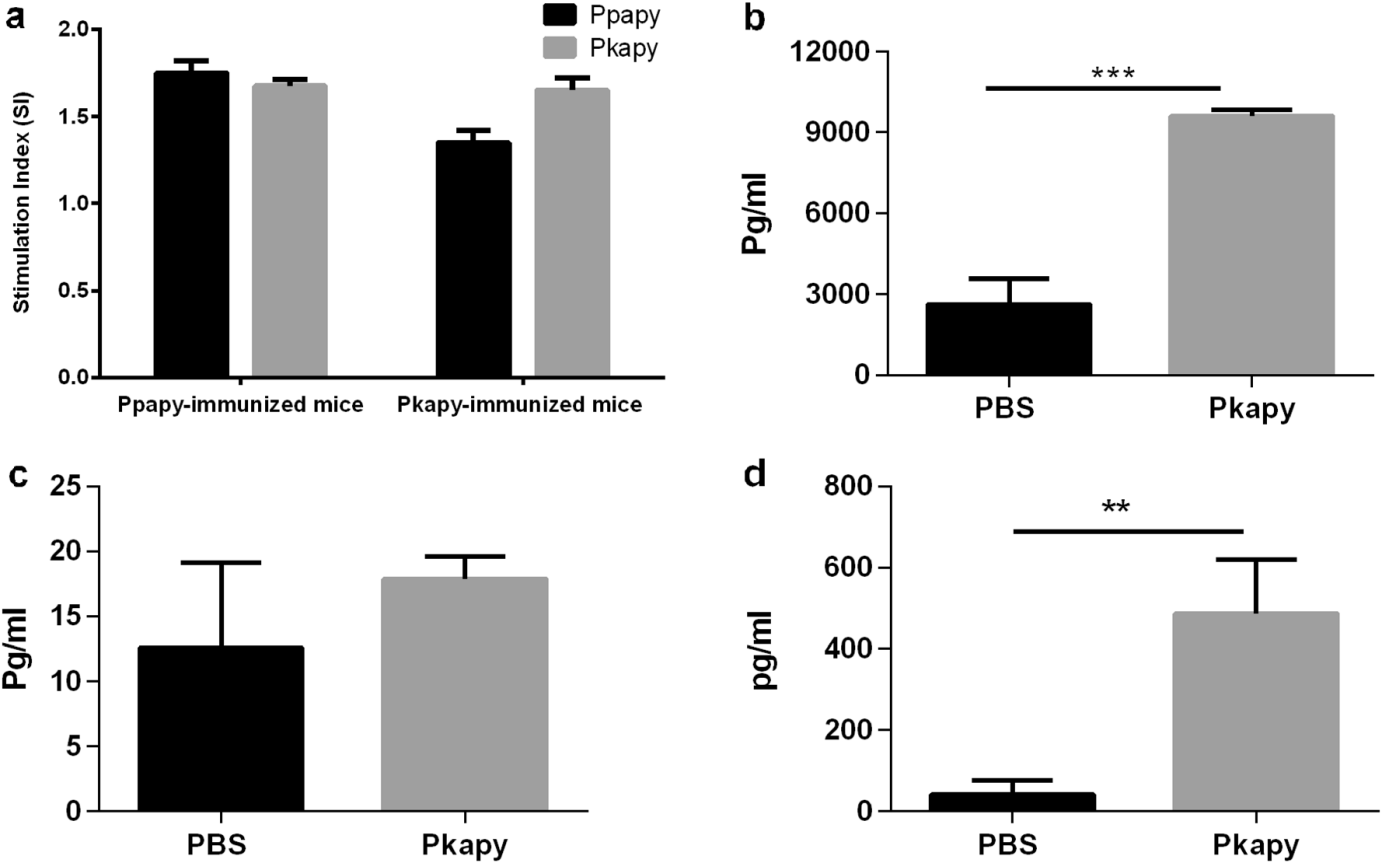
Antigen specific cell-mediated immune responses. The splenocytes from both groups of the immunized mice (n = 4 in each group) were stimulated with recombinant Pkapy, or Ppapy. (**a**) Lymphroliferative responses to Ppapy and Pkapy. Each bar indicates mean + SD of SIs. Mean + SD (pg/ml) (**b**) IFN-γ, (**c**) IL-4, and (**d**) IL-10 concentrations in the supernatants of splenocytes of Pkapy-immunized mice that were stimulated with Pkapy. (***p < 0.001; **p < 0.01).

Two weeks after the last booster, Alamar Blue was used as a sensitive test to measure the proliferation of lymphocytes in a recall response, quantitatively. The results showed that both groups of immunized mice had recall responses to the relevant recombinant apyrases. Meanwhile, no stimulation occurred with the irrelevant protein. Likewise, the splenocytes of the unimmunized mice did not respond to the recombinant apyrases. The proliferative responses of the lymphocytes from both of the immunized groups to Ppapy or Pkapy were almost similar (Fig. 5a). The concentration of secreted IFN-γ, IL-4, and IL-10 upon in vitro stimulation of the spleen cells with Pkapy was determined by ELISA. The mice immunized with Pkapy produced significant amounts of IFN-γ (*p* < 0.001, Fig. 5b), and IL-10 (*p* < 0.01, Fig. 5d), compared to the unimmunized mice. There was no statistically significant increase in IL-4 secretion by Pkapy-immunized mice (Fig. 5c).

## Discussion

VL is endemic in different parts of Iran and *P. kandelakii* is one of the most reported vectors of the disease in the North-West and North-East provinces of the country^5,6,26^. *P. kandelakii* has been found to be naturally infected with *L. infantum* in Iran and Georgia^27^. So far, salivary cDNA libraries from 9 species of *Phlebotomus* genus have been constructed and different protein families have been identified^4^; however, there is no study on the identification and characterization of salivary proteins from *P. kandelakii.* Apyrase is one of the prominent proteins of sand flies’ saliva, with the potentials as a marker of exposure and as a vaccine component against leishmaniases. To the best of our knowledge, there are two studies related to the cell-mediated responses against apyrases, as follows. It has been shown that DNA plasmid encoding *P. ariasi* salivary apyrase (*ParSP01*) induces a specific DTH response to the SGL from *P. ariasi*^21^. Furthermore, transfection of PBMC with a plasmid coding for Ppapy has resulted in a Th1 cellular immune response^22^. This designates the development of cell-mediated immune response by the apyrases and represents them as promising anti-*Leishmania* vaccine candidates. There are more studies on antibodies against apyrases; in fact, anti-apyrase antibodies have been considered as a potential biomarker of sand fly exposure and a risk factor for *Leishmania* transmission as has been shown in the case of canine exposure to *P. perniciosus* bites^15^.

In the present study, a full-length nucleotide sequence of *P. kandelakii* apyrase was characterized for the first time. Here, in contrast to many other studies that had identified the salivary proteins through the construction of cDNA libraries, we used a Genome-Walking nucleotide sequencing technique to complete the upstream and downstream sequences of a partially available middle part of *P. kandelakii* apyrase from Iran. The analysis of the coding sequence of *Pkapy* gene indicated that it belonged to the *Cimex* family of apyrases. Protein sequence alignments showed considerable homology with the other apyrases of the *Cimex* family and their vertebrate homologs, namely CAN. Bioinformatics analyses revealed that Pkapy like other known apyrases of *Phlebotomus* sand flies is a ~ 36 kDa protein. Besides, the protein is hydrophilic and stable with one *N*-glycosylation susceptible site (N247), which makes it an appropriate candidate for vaccine development.

Characterizing the diversity and degrees of homology between salivary proteins from various sand flies is an important issue*. P. papatasi* is a proven vector of *L. major* in Iran and the most abundant sand fly species in the endemic areas of the country^23^. In an attempt to identify sand fly proteins that can be used as a global or general anti-*Leishmania* vaccine, we compared the in-silico characteristics of Ppapy with those of Pkapy. Moreover, the potential antigenic cross-reactivities of these two proteins were explored. Ppapy was found to be a hydrophilic and stable molecule with two *N*-glycosylation susceptible sites (i.e., N17 and N209), compared to Pkapy which had only one *N*-glycosylation susceptible site (N247). The RMSD values of the structurally-important residues of Pkapy and Ppapy and the Ramachandran plots indicated that both proteins were structurally similar to H-CAN as the reference molecule. The superimpositions and comparisons of the critical residues of the active sites revealed that the targeted residues are completely similar to their counterparts.

The in silico analyses of the B cells epitopes showed that most of the predicted epitopes were conserved between Ppapy and Pkapy; although, Ppapy exhibits two more linear epitopes. The profiles of the antigenic peptides were very similar together since the epitopes were located in the same topological positions, based on the primary structures of both proteins. Seven discontinuous B cell epitopes with similar topological positions for each protein were also predicted.

The obtained results also indicated that the recombinant Pkapy and Ppapy proteins were immunogenic in BALB/c mice. Considering the similarities found by the bioinformatics analyses, we examined the antigenic cross*-*reactivity between Pkapy and Ppapy, by dot-blot and ELISA analyses. Cross-reactive antibodies and cross-protection have been reported between closely-related species of *Phlebotomus* subgenus *i.e. P. papatasi* and *P. duboscqi*^28^. However, *P. papatasi* and *P. kandelakii* are not closely-related species and this might be the reason for the low cross*-*reactivity between Pkapy and Ppapy. Our dot-blot data also showed that SGL from *P. papatasi* could be recognized by anti-recombinant Ppapy and Pkapy antibodies. These findings suggest the conservation of epitopes of the native apyrase in the recombinant proteins expressed in *E. coli*. This was in line with the findings that antibodies against *P. perniciosus* saliva reacted with the recombinant apyrases (i.e., rSP01 and rSP01B)^17,18,29^.

We also investigated the cellular immune response to Ppapy and Pkapy by assessment of their proliferative responses. These results indicated that splenocytes from Ppapy- and Pkapy-immunized mice could specifically recall responses to their homologous proteins. In addition, cross-reactivity was documented between Ppapy and Pkapy, since both groups of immunized mice responded to both proteins. Evaluation of the cytokines profile after stimulation with Pkapy showed higher levels of IFN-γ, in mice immunized with Pkapy, compared to the unimmunized mice. These findings indicated that the immunity to Pkapy is a Th1-dominant response. Th1 response to saliva components at the site of inoculation may activate the infected macrophages, leading to the killing of the parasites during the early phase of the infection, and may also promote a faster *Leishmania*-specific Th1 response^4^. Increased production of IL-10 by murine macrophages in response to SP01 (apyrase from *P. perniciosus* saliva) is also in line with our finding. Altogether, the immunogenic properties of Pkapy with respect to BALB/c mice highlight its potential as a component for an anti-*Leishmania* vaccine.

## Conclusions

This is the first report on the characterization of an apyrase from *P. kandelakii,* an important vector of *L. infantum* in Iran. Immunologic studies indicated that Pkapy and Ppapy are immunogenic in BALB/c mice and show cross-reactive responses. Moreover, cytokine analysis of the immunized mice revealed a Th1-type response to recombinant Pkapy. These results are providing insights into Pkapy as a potential candidate for a vector-based vaccine.

## Methods

### Sand flies

*Phlebotomus kandelakii* sand flies were captured in Bojnurd area of North Khorasan Province (North-East of Iran) using CDC traps and sticky papers. The sand flies were identified based on external and internal morphological characteristics of the head and abdominal terminalia (i.e., pharyngeal and spermathecal characteristics). The genomic DNA samples from 5 female sandflies were extracted and pooled. Female *P. papatasi* (originating from Turkey), was kindly provided by Professor Petr Volf (Department of Parasitology, Charles University, Czech Republic via infravec2; grant agreement No 731060). Salivary glands of *P. papatasi* were dissected out under a stereo microscope into PBS, and were disrupted by three freeze/thaw cycles. Salivary homogenates were centrifuged at 10,000 × *g* for 3 min and the supernatants were used for the experiments.

### Determination of the 5′- and the 3′-end sequences of *P. kandelakii apyrase* gene by Genome-Walking method

Since at the beginning of this study, only a partial middle part of *Pkapy* gene was publicly available, a Genome-Walking sequencing technique was performed to obtain the upstream and downstream sequences of the available segment, known as *P. kandelakii* clone kandG2a apyrase-like protein (GenBank accession N^o^.: JF899992). As shown in Table 1a, gene-specific primers (i.e., Forwards: F-GSPa, F-GSPb, and F-GSPc; Reverses: R-GSPa, R-GSPb, and R-GSPc) were designed and synthesized (Cinnaclon, IR-Iran) according to the 3′-end and the 5′-end sequences of JF899992 fragment using Gene Runner software (version 5.1.06 Beta). The specificity of the primers for PCR was checked by nucleotide BLAST on NCBI.

Seven Genome-Walking primers (i.e., GWPs A-G), as well as long and short universal tagging primers (i.e., UAP-N1, UAP-N2), were used according to the previous studies^24,30^. Firstly, to amplify the targeted sequences downstream of the gene, the synthesis of single-stranded DNA (ssDNA) molecules was evaluated using F-GSPa primer at 57–65 °C using a thermocycler (Mastercycler gradient 5331, Eppendorf, Germany). The products were electrophoresed on 1% agarose gel and analyzed. The lowest temperature in which no amplicon was seen was selected as the best temperature for further ssDNA molecule synthesis. The synthesis was performed separately in 7 tubes labeled A to G. Each tube was allocated for one of the GWPs in the following step. Amplification reactions were composed of 7.5 μl ExPrimeTaq Premix 2X (Genet Bio, South Korea), 400 nM F-GSPa primer, and sterilized D.W. up to 15 μl. The PCR program is mentioned in Table 1b.

Secondly, after immediately adding 1 μl from each of the 7 GWPs, along with 3 μl ExPrimeTaq Premix and 1 unit of Taq DNA polymerase (Genet Bio, South Korea) individually to the 7 reaction tubes, PCR was carried out according to the program indicated in Table 1b. Thirdly, 1 μl of 25-fold diluted PCR products was used as a template with UAP-N1 and F-GSPb primers for the first nested PCR as mentioned in Table 1b. Fourthly, the previous PCR products were diluted (25-fold) and 1 μl of each reaction was used as templates for the second nested PCR with F-GSPc and UAP-N2 primers, performed each time in 7 separate tubes using the same above-mentioned program (Table 1b). The amplicons of the final step were evaluated by 1.5% agarose gel electrophoresis.

The fragments with acceptable sizes were subjected to gel extraction and were then subcloned into a PTZ57R/T (Thermo Scientific InsTAclone PCR Cloning Kit, USA) and sequenced (Macrogen, South Korea). To identify the upstream sequence of the gene, all the above-mentioned procedures were carried out, except that the specific reverse primers were used.

### In silico analyses of the sequence

The obtained full sequence of the *Pkapy* gene was subjected to BLASTX <http://blast.ncbi.nlm.nih.gov/Blast.cgi> which indicated that the sequence belonged to the *Cimex* family of apyrases. The protein sequence of Pkapy was aligned with other apyrase protein sequences from *P. papatasi* (AAG17637.1), *P. orientalis* (AGT96454.1), *Lutzomyia (Lu.) longipalpis* (AAD33513.1), *Cimex lectularius* (AAD09177.1), *Drosophila melanogaster* (CAL26008.1), and their mammalian homologous (i.e., calcium-activated nucleotidases (CANs) from *Rattus norvegicus* (CAC85467.1), *Canis lupus familiaris* (XP_005624095.1) and Human (Q8WVQ1.1)). Multiple alignments were performed using Clustal Omega (https://www.ebi.ac.uk/Tools/msa/clustalo/) based on the ClustalW method^31^. The presence and location of signal peptide cleavage sites were predicted by Signal P-5.0 server (http://www.cbs.dtu.dk/services/SignalP/) based on a combination of several artificial neural networks^32^. The physicochemical properties such as instability index, isoelectric point, aliphatic index, grand average of hydropathy (GRAVY), molecular weight (Mw), and isoelectric point (pI) were analyzed with ProtParam server (https://web.expasy.org/protparam/). To predict the amino acids that undergo glycosylation, NetNGlyc 1.0 (http://www.cbs.dtu.dk/services/NetNGlyc/) and NetOGlyc 4 servers (http://www.cbs.dtu.dk/services/NetOGlyc/) were used. The secondary structure was predicted by PHD secondary structure server (https://npsa-prabi.ibcp.fr/cgi-bin/secpred_phd.pl).

### Tertiary structure prediction and superimposition

SWISS-MODEL server (http://swissmodel.expasy.org) was employed to predict the 3D structure of Pkapy and Ppapy based on homology modeling. According to QMEAN (Qualitative Model Energy Analysis) and GMQE (Global Model Quality Estimation), the best-predicted structures with the highest score for each protein were selected and used for comparing the structural properties. The superimposition and root-mean-square deviation (RMSD) analyses were performed by DeepView/Swiss-PdbViewer v.4.1.0 and UCSF Chimera v.1.11.2^33^.

### Prediction of B cell epitopes

Linear B cell epitopes of Pkapy and Ppapy were predicted using BepiPred-2.0 of the IEDB analysis resource (http://tools.iedb.org/bcell/). The BepiPred-2.0 server predicts B-cell epitopes from the protein sequence, where the residues with scores above the default value of 0.5 were predicted as epitopes and colored in yellow on the graph. Further, the discontinuous B cell epitopes were predicted using Ellipro method in the IEDB database (http://tools.iedb.org/ellipro/) which is based on solvent accessibility and flexibility^34^.

### Cloning, expression, and purification of recombinant Pkapy and Ppapy

Constructs containing regions encoding each protein without the putative secreted signal peptide were optimized for expression in *E. coli* and synthesized by Biomatik Corp. (Canada), subcloned into pET-21b (+) vector for expression with a C-terminal 6 × His-tag*.* The constructed expression vectors were transformed into *E. coli* BL21 (DE3; Invitrogen, USA) and verified by restriction digestion and nucleotide sequencing. Protein expression was induced by the addition of Isopropyl β-d-1-thiogalactopyranoside (IPTG) to a final concentration of 0.5 mM to a culture of LB with ampicillin (100 μg/ml), containing verified *E. coli* BL21 with the construct, grown to OD_600_ ~ 0.5 (37 °C, 200 RPM). The culture was grown for an additional 2 h after the induction as above.

The expressed recombinant proteins were purified by Ni–NTA Superflow resin (Qiagen, Germany), following the instructions under denaturing conditions. The proteins were dialyzed overnight against several changes of PBS and endotoxins were removed using a Pierce High-Capacity Endotoxin Removal Resin spin column (Thermo Scientific, USA), according to the manufacturer’s recommendations. The concentrations of the purified recombinant proteins were determined by Bradford assay^35^.

### SDS-PAGE and Western blotting analyses

The purified recombinant Ppapy and Pkapy proteins were subjected to 10% SDS-PAGE, then electroblotted onto PVDF membrane using a wet Bio-Rad transfer system (Bio-Rad, Hercules, USA). The membrane was blocked with 5% (w/v) skimmed milk in PBS and incubated with anti-His antibody (Qiagen, Germany) as the primary antibody. Finally, the membrane was incubated with goat anti-mouse IgG HRP antibody (Sigma, Germany) as the secondary antibody. The reactivity was detected using 3, 3-diaminobenzidine tetrahydrochloride (DAB).

### Immunization with the recombinant apyrases

Female BALB/c mice (4–6 weeks old) were purchased from the animal facility of the Production Complex of the Pasteur Institute of Iran. All animal studies were performed in line with the ARRIVE reporting guidelines. Approval was granted by the Ethics Committee of the Pasteur Institute of Iran (IR.PII.REC.1395.109), and the experiments were performed in accordance with relevant guidelines and regulations.

Mice (4 in each group) were immunized three times at 2-week intervals subcutaneously at the base of the tails with 10 µg of either Ppapy or Pkapy along with 15 µg Quil A (Invivogen, USA), as an adjuvant. The optimal dose of proteins for immunization was determined in a preliminary study in which mice were immunized with 5, 10, and 15 µg of Pkapy. When the sera were checked for the presence of anti-apyrase antibody, the results of 15 µg Pkapy were almost similar to 10 µg; hence, the latter amount was selected for the immunization. The control group received Quil A only. Mice were retro-orbitally bled for sera preparation two weeks after the last immunization.

### Dot‐blot immunoassay and ELISA

To detect immunogenicity and the potential cross-reactivity of the recombinant apyrases, dot-blot and ELISA were performed. For the dot-blot assay, 1 μg of each Ppapy, Pkapy, SGL (1 gland/dot) of female *P. papatasi*, and an unrelated protein (i.e., BSA as a negative control) were spotted onto a nitrocellulose membrane. The spots were dried and the membrane was blocked with 5% skimmed milk in PBS. The membrane was then incubated with 1:10 diluted sera collected from the immunized and unimmunized mice for 2 h at RT. For the secondary antibody, an HRP-conjugated goat anti-mouse antibody was added and incubated at RT for 1 h. Finally, spots were developed using DAB substrate solution in presence of hydrogen peroxide. Stained dots on a white background indicated positive results.

Ppapy, Pkapy, and BSA were used in 1 μg/well concentration to coat 96-well ELISA plates (Greiner, Germany) in duplicates and were then incubated at 4 °C overnight. After washing, pre-diluted sera (1:100) from each mouse (4 mice in each group) were added to the wells in a direct and cross manner to evaluate its reactivity with the immunized protein and the counterpart protein. After incubation for 2 h at RT, HRP-conjugated goat anti-mouse antibody (Sigma, Germany) was added to the plates and incubated for 1 h at RT. The plates were developed with 3,3′,5,5′-Tetramethylbenzidine (TMB; Sigma, Germany) and read at 450 nm by a microplate reader (Biochrom Anthos 2020, UK). The levels of IgG antibody were reported as OD values of each well minus the OD values of the control wells.

### T-cell proliferation and cytokine assay

Alamar blue assay was used to evaluate the proliferative responses of the lymphocytes to the recombinant proteins. Two weeks after the last immunization, the spleen cells from each mouse (4 mice in each group) were recovered and cultured as previously described^36^. In brief, the splenocytes were stimulated with recombinant Pkapy, Ppapy, or an irrelevant recombinant protein (FHA from *Bordetella pertussis*). Moreover, the splenocytes of unimmunized mice were stimulated with the recombinant apyrases. Negative control cultures consisted of the medium without any antigen. The cultures were incubated at 37 °C, 5% CO_2_ for 3 days. Subsequently, 20 μl of Alamar blue reagent (Sigma, Germany; 0.15 mg/ml in PBS) was added to each well and incubated for 4 h. Three replicates of each sample were analyzed on a microplate reader at 570 nm with a reference wavelength of 690 nm (BioTek ELx808 Absorbance Microplate Reader, USA) and the percentage of Alamar Blue reduction was calculated for stimulated and unstimulated cells according to Al-Nasiry et al*.*^37^. The stimulation index (SI) was calculated by dividing the mean results of the stimulated cells by the mean results of the un-stimulated cells. For cytokine assays, splenocytes from Pkapy-immunized mice were stimulated with recombinant Pkapy for 4 days and subsequently, the culture supernatants were recovered and stored at − 80 °C for determination of IFN-γ, IL-4, and IL-10 levels. Cytokines were assessed by Mabteck ELISA kits (Stockholm, Sweden), according to the manufacturer’s instructions.

### Statistical analysis

ANOVA followed by Tukey’s multiple comparisons test and Student’s t-test were used to evaluate the statistical significance of the obtained data for ELISA and T cell proliferation, respectively. Statistical analysis was performed with GraphPad Prism software (Prism 8.0.2., 2019, San Diego, CA). The *p* < 0.05 was considered to be significant, and data were represented as mean + SD.

## Supplementary Information

Supplementary Information.

## Data Availability

The datasets generated and analyzed during the current study are available in the repository, NCBI GenBank repository, Accession N^o^.: MN893300.

## Abbreviations

Pkapy: *Phlebotomus kandelakii* Apyrase
Ppapy: *Phlebotomus papatasi* Apyrase
SGL: Salivary gland lysate
DTH: Delayed-type hypersensitivity
GSPs: Gene-specific primers
GWPs: Genome-Walking primers
UAPs: Universal tagging primers
ssDNA: Single-stranded DNA
pI: Isoelectric point
GRAVY: Grand average of hydropathy
QMEAN: Qualitative model energy analysis
GMQE: Global model quality estimation
RMSD: Root-mean-square deviation
IEDB: Immune Epitope Database
IPTG: Isopropyl β-d-1-thiogalactopyranoside
SDS-PAGE: Sodium dodecyl sulphate–polyacrylamide gel electrophoresis
ELISA: Enzyme-linked immunosorbent assay
PVDF: Polyvinylidene fluoride
DAB: 3,3-Diaminobenzidine tetrahydrochloride
TMB: 3,3′,5,5′-Tetramethylbenzidine
SI: Stimulation index
CANs: Calcium-activated nucleotidases
BSA: Bovine serum albumin
HRP: Horseradish peroxidase
VL: Visceral leishmaniasis
CL: Cutaneous leishmaniasis
PDB: Protein data bank
