# Supplementary material for: Molecular identification of Phlebotomus kandelakii apyrase and assessment of the immunogenicity of its recombinant protein in BALB/c mice
Source: Sci Rep. 2023 May 30;13:8766. doi: 10.1038/s41598-023-36037-z (PMC10228885; doi:10.1038/s41598-023-36037-z)
Supplement: Supplementary file 1 — Supplementary Information. [file 41598_2023_36037_MOESM1_ESM.pdf]

Molecular identification of *Phlebotomus kandelakii* apyrase and assessment of the immunogenicity of its recombinant protein in BALB/c mice

Shima Fayaz[1,2], Abbasali Raz[3], Fariborz Bahrami[1], Pezhman Fard-Esfahani[2], Parviz Parvizi[4], Soheila Ajdary[1]*

[1]Department of Immunology, Pasteur Institute of Iran, Tehran, Iran

[2]Department of Biochemistry, Pasteur Institute of Iran, Tehran, Iran

[3]Malaria and Vector Research Group (MVRG), Biotechnology Research Center (BRC), Pasteur Institute of Iran, Tehran, Iran

[4]Department of Parasitology, Pasteur Institute of Iran, Tehran, Iran

* **Correspondence**:

Soheila Ajdary,

Immunology Department,

Pasteur Institute of Iran,

69 Pasteur Ave., Tehran 13169-43551, IR-Iran

Tel and fax: +98 21 64 11 28 40

E-mail: sohary@yahoo.com

ajdsoh@pasteur.ac.ir

Figure S1. Downstream and upstream characterization of *P. kandelakii* apyrase gene sequence (a) 3′ end characterization: PCR with combination of F-GSPc and UAP-N2 primers with F-GSPb and UAP-N1 products as templates in GWA to G tubes. Three amplicons of GWA (*i.e.* two amplicons of GWF and GWG) were selected for further analysis. (b) 5′ end characterization: PCR with R-GSPb and UAP-N1 primers and using R-GSPc and UAP-N2 as templates in all GW tubes. Only two amplicons of GWA were submitted for the next analysis. The DNA ladder is 1000-bp. NC implies no template negative control

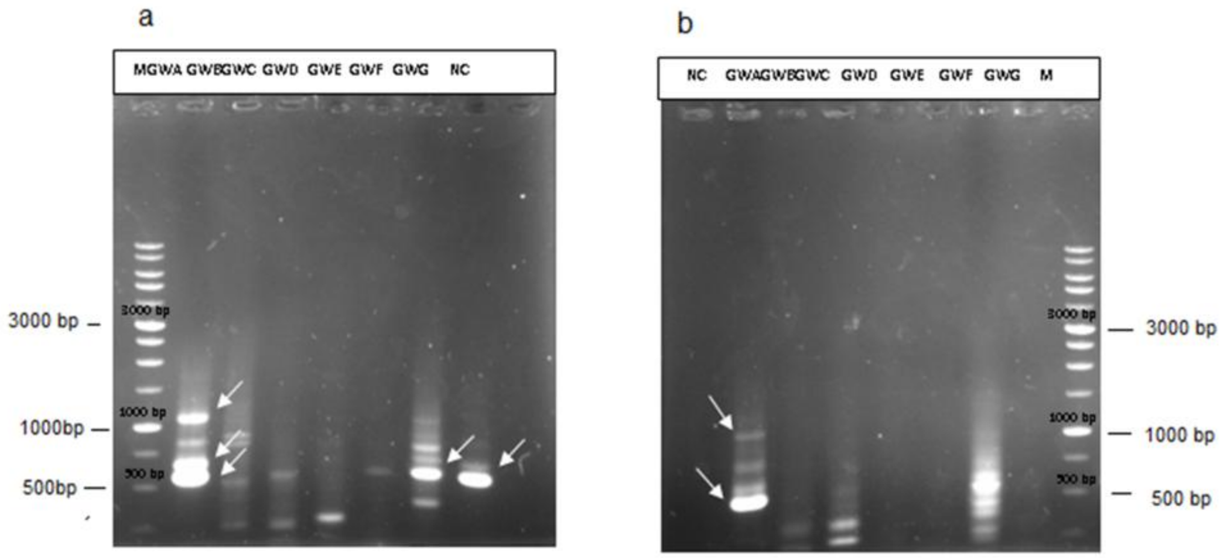

Figure S2. Graphical linear epitopes results of Pkapy (a) and Ppapy (b). Residues with scores above the threshold (0.5; colored in yellow) are predicted as epitope.

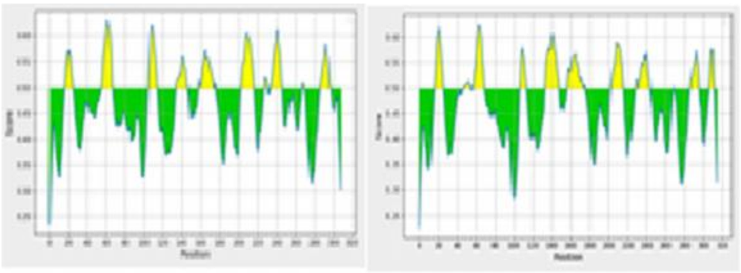

Figure S3. a) SDS-PAGE gel stained with Coomassie Brilliant Blue. Ni-NTA purified proteins: lanes 1, 2 Ppapy, lanes 3, 4 Pkapy. Lane 5 MW marker, lane 6 unpurified Ppapy, lane 7 unpurified Pkapy. b) Western blotting of purified Ppapy lanes 1, 2, Pkapy lane 3, MW marker lane 4, incubated with anti-His antibody

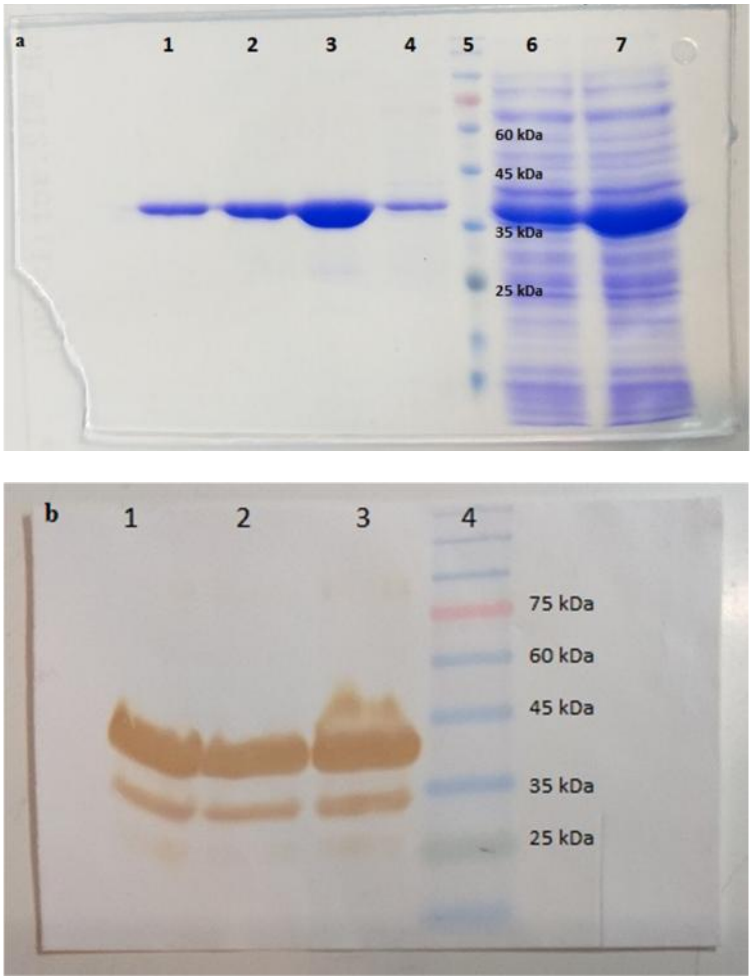

S1 Table. Discontinuous antigenic epitopes of Pkapy and Ppapy

Discontinuous Epitope*	Residues**	Number of residues	Score
KDE1	A:S50, A:E51, A:N52, A:L53, A:H54	5	0.76
KDE2	A:I21, A:K22, A:D23, A:D24, A:K25, A:N26, A:R27, A:F28, A:F56, A:T57, A:Q58, A:N59, A:S60, A:Y61, A:H62, A:G63, A:L64, A:G65, A:E84	19	0.753
KDE3	A:A134, A:F135, A:L136, A:D137, A:A138, A:K139, A:T140, A:M141, A:N142, A:I143, A:D144, A:R145, A:Y163, A:E165, A:N166, A:K169, A:N173, A:A174, A:M175, A:G176, A:I177, A:T178, A:S179, A:G180, A:F181, A:S204, A:N205, A:Q206, A:Q207, A:F208, A:T209, A:A210, A:R211, A:I212,A:N225, A:E226, A:N227, A:F228, A:S229, A:D230, A:V231, A:K232, A:A233	43	0.719
KDE4	A:T5, A:K6, A:H39, A:N40, A:G41, A:E42, A:K43, A:Y44, A:S190, A:P191, A:R192, A:K193, A:N194, A:N219, A:I236, A:D237, A:K238, A:A239, A:A240, A:Q241, A:I253, A:P254, A:G255, A:T256, A:K257, A:N258, A:N259, A:D281, A:I282, A:T283, A:G284, A:K285, A:T286, A:L287, A:L288	35	0.686
KDE5	A:N75, A:G76, A:K77,A:E90, A:K92, A:H93, A:G94, A:G95, A:N96, A:I98, A:P99, A:W100, A:L103, A:K104, A:N105, A:D106, A:D107, A:G108, A:N109, A:Q110, A:K111, A:D112, A:G113, A:N123, A:D124, A:K125, A:S154, A:E155, A:S156, A:G157, A:H158, A:I159, A:T160, A:N161	34	0.68
KDE6	A:F7, A:I8, A:P9, A:G35, A:E36, A:K38, A:T45, A:L46, A:S47, A:I48, A:R49,A:F304	12	0.674
KDE7	A:E214, A:N215, A:T216, A:G217, A:D242, A:P243, A:A244, A:S245, A:R269, A:N270, A:G271, A:Q272	12	0.564
PDE1	A:K50, A:K51, A:E52, A:D53, A:H54	5	0.779
PDE2	A:I21, A:S22, A:P23, A:K24, A:N25, A:D26, A:N27, A:N28, A:Y29, A:F57, A:T58, A:K59, A:G66	13	0.822
PDE3	A:S135, A:F136, A:T137, A:D138, A:K139, A:T140, A:G141, A:K142, A:L143, A:N144, A:S145,A:K165, A:E166, A:D169, A:K172, A:S173, A:A174, A:M175,A:I177, A:P178, A:N179, A:G180, A:F181, A:C203, A:S204, A:E205, A:R206, A:P207, A:F208, A:D209, A:T210, A:K211, A:T212, A:E214, A:T215, A:I216, A:G217, A:N219, A:S225, A:E226, A:N227, A:F228, A:E229, A:I230, A:I231, A:K232, A:K237, A:G238, A:K239, A:S240, A:I241, A:N242, A:R243, A:A244, A:K269, A:D270, A:D271, A:K272	58	0.679
PDE4	A:G5, A:T6, A:I7, A:Y8, A:N9, A:G36, A:E37, A:L38, A:I39, A:E40, A:V41, A:G42, A:D43, A:K44, A:Y45, A:S46, A:V47, A:K48, A:M49, A:L192, A:K193, A:I236, A:L253, A:P254, A:D255, A:S256, A:D257, A:D258, A:Q259, A:D281, A:I282, A:T283, A:G284, A:R285, A:V286, A:L287, A:M288, A:P289, A:E290, A:L303, A:L304	41	0.673
PDE5	A:K76, A:K78, A:W101,A:A105, A:N106, A:G107, A:N108, A:G109, A:D110, A:Q111, A:T112, A:D113, A:G114, A:G124, A:D125, A:K126, A:D154, A:Q155, A:D156, A:G157, A:K158, A:V159, A:Q160, A:S161, A:L162, A:D163	26	0.675
PDE6	A:Y60, A:A61, A:Y62, A:K63, A:G64, A:R65, A:K85, A:S86	8	0.572
PDE7	A:R91, A:K93, A:T94, A:N95, A:A96, A:D97, A:I99, A:P100	8	0.714

KDE: kandelakii* apyrase discontinuous epitope, PDE: *papatasi* apyrase discontinuous epitope

** Position of epitopes is considered after signal peptide exclusion.
